# Perfluorochemical Liquid-Adenovirus Suspensions Enhance Gene Delivery to the Distal Lung

**DOI:** 10.1155/2011/918036

**Published:** 2011-08-18

**Authors:** Jeffrey A. Kazzaz, Marlene S. Strayer, Jichuan Wu, Daniel J. Malone, Hshi-chi Koo, Thomas H. Shaffer, Jonathan M. Davis, David S. Strayer, Marla R. Wolfson

**Affiliations:** ^1^The Cardiopulmonary Research Institute and Departments of Medicine and Pediatrics, SUNY Stony Brook School of Medicine, Winthrop University Hospital, Mineola, NY 11507, USA; ^2^Department of Pathology, Thomas Jefferson University Medical Center, Philadelphia, PA 19107, USA; ^3^Departments of Physiology and Pediatrics, Temple University School of Medicine, 3420 North Broad Street, Philadelphia, PA 19140, USA; ^4^Nemours Research Lung Center, Alfred I. DuPont Hospital for Children, Wilmington, DE 19803, USA; ^5^Department of Pediatrics, The Floating Hospital for Children at Tufts Medical Center, Boston, MA 02111, USA; ^6^Center for Inflammation, Translational, and Clinical Lung Research (CILR) and Temple Lung Center, Temple University School of Medicine, Philadelphia, PA 19140, USA

## Abstract

We compared lung delivery methods of recombinant adenovirus (rAd): (1) rAd suspended in saline, (2) rAd suspended in saline followed by a pulse-chase of a perfluorochemical (PFC) liquid mixture, and (3) a PFC-rAd suspension. Cell uptake, distribution, and temporal expression of rAd were examined using A549 cells, a murine model using luciferase bioluminescence, and histological analyses. Relative to saline, a 4X increase in transduction efficiency was observed in A549 cells exposed to PFC-rAd for 2–4 h. rAd transgene expression was improved in alveolar epithelial cells, and the level and distribution of luciferase expression when delivered in PFC-rAd suspensions consistently peaked at 24 h. These results demonstrate that PFC-rAd suspensions improve distribution and enhance rAd-mediated gene expression which has important implications in improving lung function by gene therapy.

## 1. Introduction

Gene delivery to distal lung epithelium has proven difficult because of the extensive system of large and small airways, mucociliary clearance, and the presence of dense glycocalyx lining the airway lumen. Lung surfactant acts as a barrier and surfactant proteins act as collectins to remove recombinant proteins and viruses for gene therapy [1, 2]. The distribution of the coxsackie-adenovirus receptor (CAR) on basolateral surfaces of cells has led to the proposed use of reagents that disrupt tight junctions and improve transduction of airway epithelial cells [3–5]. As an alternative approach to improve distribution, several studies have used an intratracheal pulse-chase method in the lung to deliver recombinant adenovirus (rAd) and adeno-associated virus vectors in a small volume of saline administered followed by a larger volume of perfluorochemical (PFC) liquid [3, 6–11]. These studies have demonstrated increased total lung gene expression, distribution of gene expression, and enhanced delivery to the distal lung over saline alone. However, uniform distribution with increased alveolar uptake has not been demonstrated consistently. In addition, efficacy of these techniques with respect to differences in overall expression over time has not been elucidated.

PFC liquids are synthetically produced fluorinated hydrocarbons that are inert, capable of carrying dissolved gasses, and dense enough to open areas of collapsed lung. PFC liquids have long been proposed as a ventilatory treatment as well as a method to homogeneously deliver bioactive agents to the lung. Studies of liquid ventilation in animal models, and clinical settings of acute lung injury demonstrate that PFC liquids have a number of salutary effects in injured lungs. Following liquid ventilation, animals with experimentally induced acute lung injury demonstrate improved gas exchange, lung compliance, less hemorrhage, edema, inflammation, and oxidation compared to similarly injured animals undergoing conventional gas ventilation [12–20]. Histopathologic injury and bronchoalveolar lavage (BAL) fluid content of total white cells, neutrophils, and proinflammatory cytokines were reduced in animals or patients with acute lung injury undergoing liquid ventilation compared to gas ventilation [19, 21]. These effects have been attributed to both mechanoprotective and cytoprotective mechanisms [22, 23]. Several *in vitro* studies have demonstrated that intracellular uptake of PFC alters behavior of alveolar epithelial and inflammatory cells, particularly neutrophils and monocytes/macrophages, contributing to anti-inflammatory properties [24–27]. However, physicochemical properties of PFC liquids such as viscosity, vapor pressure, and lipid solubility vary depending upon the chemical composition and arrangement of carbon-fluorine bonds. These characteristics are important determinants of the degree to which PFC liquids distribute throughout the lung, evaporate from the lung, and move across cell membranes [14, 25, 28–31]. 

With respect to optimal physiological profiles and anti-inflammatory properties, the most therapeutic PFC liquids have relatively high viscosity and low vapor pressure [13, 15]. These characteristics allow the PFC liquid to resist redistribution and rapid elimination. These PFC liquids tend to remain where they are initially administered, and additional doses are less likely to be required [14]. A recent, novel approach has been to combine different PFC liquids to engineer a desired viscosity and vapor pressure. A systematic comparison of the anti-inflammatory properties of a number of PFC liquids in an *in vivo* model demonstrated that a 1 : 3 ratio of PP2 and PP9 had the greatest anti-inflammatory impact on the lung [13, 15].

In this paper, we used a mixture of PP2:PP9 as a gene delivery vehicle using rAd both *in vitro* and* in vivo* and compared various methods of delivery: (1) rAd suspended in saline, (2) rAd suspended in saline followed by a pulse-chase of PFC liquid, and (3) rAd as a PFC liquid suspension. To test the hypothesis that rAd delivered as a PFC suspension would result in higher levels of transgene expression in lung parenchyma, we examined the expression in A549 cells. *In vivo* studies in a murine model using rAd-LacZ construct demonstrated a more uniform distribution than when delivered in saline or by the pulse-chase method. The effect of this methodology on temporal expression *in vivo* was determined using a rAd-luciferase construct in a murine model. These data demonstrate that use of PFC suspensions for the delivery of rAd increases expression in the lung periphery and gives a more reliable temporal pattern of expression.

## 2. Methods

### 2.1. rAd Constructs

Replication-deficient type 5 adenovirus encoding LacZ (rAd-CMVntLacZ), was obtained from the Gene Transfer Vector Core at the University of Iowa [32]. The recombinant adenovirus encoding the firefly luciferase gene (rAd-CMVLuc) was obtained from the Vector Core Facility at the University of Pittsburgh.

### 2.2. PFC-rAd Suspensions

The perfluorochemical used in this study was 25% perfluorocyclohexane [PP2]/75% perfluoromethyldecalin [PP9] obtained from F2 Chemicals, Ltd, Lancashire, UK. PFC suspensions were prepared using a modified method described previously to ensure stability and homogenous distribution [33, 34]. Briefly, rAd constructs in saline (2.7 × 10^10^ viral particles in 2 mL/kg) and the PFC fluid (25% PP2/75% PP9; 10 mL/kg) were sonicated (Branson 2510, Danbury, CT) for 5–10 minutes. 

### 2.3. rAd Transduction Efficiency *In Vitro*

Human alveolar epithelial A549 cells (American Type Culture Collection CCL-185) were grown in Ham's F-12-K medium (GIBCO BRL, Life Technologies, Gaithersburg, MD) supplemented with 10% heat-inactivated fetal bovine serum, 2 mM glutamine, 100 U/mL penicillin, and 100 *μ*g/mL streptomycin. Cells (4 × 10^5^) were maintained at 37°C in 95% room air-5% CO_2_ in a humidified chamber, seeded on a 6-well plate containing coverslips and allowed to adhere overnight. Subconfluent A549 cells were incubated with rAd at a multiplicity of infection (MOI) of 100 viral particles/cell in 0.6 mL of complete media or PFC liquid on a rocking platform for 2–48 h at 37°C. For incubations longer than 8 h, 0.6 mL of media was added to the PP2:PP9 wells to prevent the PFC liquid from evaporating and thus protecting the cells from dehydration. At the end of the transfection period, PFC liquid was siphoned off, and cells were refreshed with medium. 

To quantify transduction efficiency in culture, assessment of *β*-galactosidase (*β-*gal) activity was performed 48 h following transduction on near confluent cells grown on coverslips as per manufacturer's instructions (Sigma Chemicals, St. Louis, Mo). Briefly, cells were rinsed with phosphate-buffered saline (PBS), and then fixed in 0.1% glutaraldehyde for 5 min at room temperature. After three washes with PBS, cells were stained with 1 mg/mL X-gal (Invitrogen) for 2 h at 37°C. In transfected cells, *β-*gal cleaved X-gal to produce a blue stain. Coverslips were then washed and mounted on microscope slides for viewing. Ten consecutive fields were captured using a Nikon TL300 inverted microscope equipped with a Sony 3CCD Progressive camera, a PC computer, and Adobe Photoshop version 7.0 with the appropriate import plugin using identical settings. Images were then viewed in Metamorph (Molecular Dynamics), and X-gal positive cells were counted manually. 

### 2.4. Instillation of rAd *In Vivo*

Spontaneously breathing, C57BL/6 and Balb/c mice (6–8 wks; 15–20 gm) randomly assigned to vehicle and time-sequenced groups and then anesthetized by intraperitoneal injection (ketamine: 40 mg/kg; xylazine: 8 mg/kg). Once sedated, local anesthesia was applied (lidocaine), and the trachea was isolated through a superficial incision. Instillation was performed during spontaneous breathing by tracheal puncture using a 0.50 mL syringe with 29 G needle, approximately 2 cartilaginous rings below the cricoid. Following instillation, animals were rotated consistently to augment distribution until the mice demonstrated normal motor activity and grooming (i.e., within 30 min). All procedures were approved by the Institutional Animal Care and Use Committee at Temple University School of Medicine and were in accordance with National Institutes of Health guidelines.

### 2.5. rAd Transduction Efficiency *In Vivo*

Spatial distribution of gene expression was assessed in C57BL/6 mice (*n* = 9) that received rAd-CMVLacZ in saline (2.7 × 10^10^ viral particles in 2 mL/kg), rAd-CMVLacZ in saline (2 mL/kg) followed by PFC (10 mg/kg) (pulsed chase method), or rAd-CMVLacZ in saline (2 mL/kg) suspended in PFC (10 mL/kg) (PFC suspension). Additional animals (*n* = 6) instilled with saline alone (2 mL/kg) or PFC alone (10 mL/kg) were used as controls. Temporal distribution of gene expression was assessed by bioluminescence in Balb/c mice (*n* = 8) that received rAd-Luciferase in saline (10^10^ or 5 × 10^10^ viral particles) suspended in PFC (2 mL/kg). 

### 2.6. Spatial Distribution

In the lungs of these animals, spatial distribution was assessed 48 h following intratracheal administration. The trachea was cannulated, and the lungs were pressure-clamped at 15 cm H_2_O, vascularly perfused with cold Millonig's buffer, and removed en bloc. Lungs were then fixed in glutaraldehyde, incubated in X-gal reaction solution (1 mg/mL of X-gal) at 37°C for 4 h, and postfixed in formalin. Lung mounts were then paraffin embedded for imaging and preparation for histological analysis. The paraffin-embedded tissue blocks of lungs were viewed on a Nikon SME10 dissecting microscope; images were captured with a Nikon E5000 Coolpix camera and imported into Adobe Photoshop for digital output. Thin sections (5 *μ*m) were mounted on slides, stained with hematoxylin (H) and eosin (E), and examined by light microscopy. Tissue sections were viewed on a Nikon Optishot microscope; images were captured with a Spot Insight camera and imported into Image ProPlus for digital output. 

### 2.7. Temporal Gene Expression

Temporal gene expression of the rAd-CMV vector was assessed on days 1, 2, 4, 7 and, 10 following administration of the rAd encoding the firefly luciferase gene (rAd-CMVLuc). This bioluminescent assay system is used to indirectly measure a gene of interest where the luciferase gene is placed downstream of the relevant promoter. The substrate, luciferin, reacts with oxygen in the presence of the enzyme luciferase, resulting in the formation of light. *In vivo *luciferase expression was visualized in anesthetized Balb/c mice (*n* = 3-4 animals/group). Animals were administered 100 *μ*L (150 mg/kg) of luciferin by intraperitoneal (IP) injection, placed under gas anesthesia (isoflurane), and imaged 15–20 min after administration with the IVIS 50 imaging system (Xenogen Corp., Alameda, Callif) and the Living Image software package (Caliper Life Sciences, Hopkinton, Mass). An initial dose-response curve determined that signal peaked in the lung at 15 min and began to subside at 25 min (data not shown). Image settings of a 3 min exposure at high sensitivity (8 × 8 binning) were used except when these settings resulted in a saturated image. The images were quantified using identical region sizes. Background values were determined from sham-treated animals that were delivered IP luciferin in an identical manner. 

### 2.8. Statistical Analyses

For viral transduced cells, a Student's *t*-test was performed comparing groups within each time period. Differential *β*-gal and luciferase expression in the lungs was compared using one-way ANOVA with a Fisher- or Bonferroni-adjusted posthoc comparison of means between groups. All analyses were performed using the SAS software package version 8.1 (SAS Institute, Cary NC, 2001). Results are reported as mean ± standard deviation. 

## 3. Results

### 3.1. PP2:PP9 Improves rAd Transduction Efficiency In Vitro

**Figure 1:**
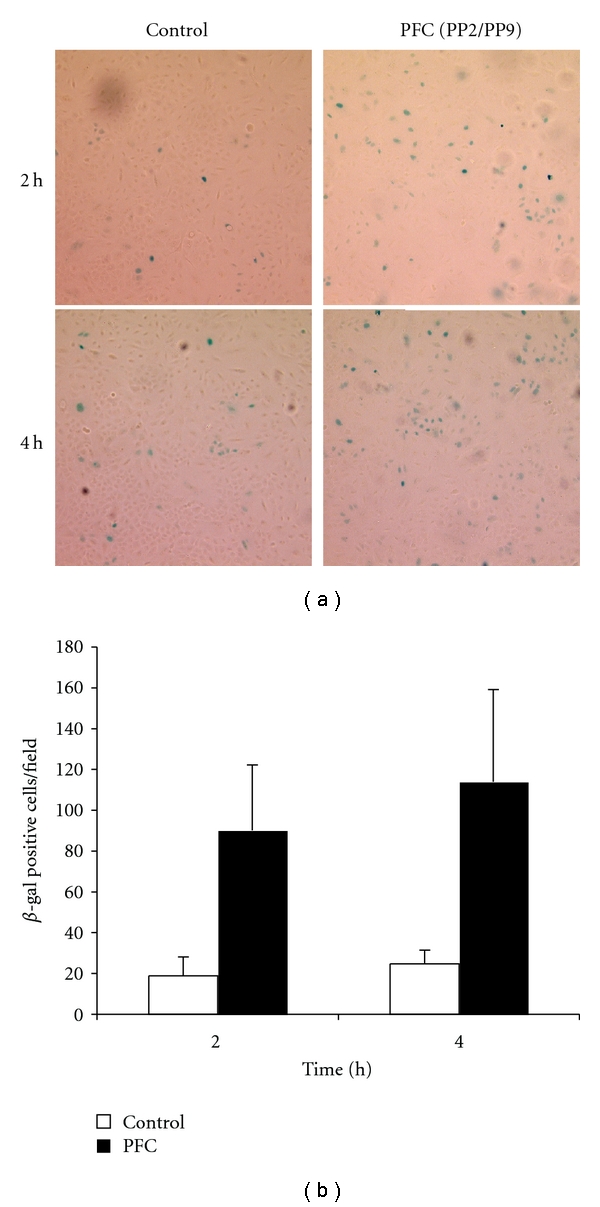
PFC liquid increases transduction efficiency in cell culture. (a) Representative fields of A549 cells transduced with rAd-CMVLacZ (MOI = 100 viral particles/cell) in either saline (Control, left) or PFC liquid (right) for 2 h (top) or 4 h (bottom) followed by X-gal staining. (b) Consecutive low-power fields were acquired under identical settings, and *β*-gal positive cells were enumerated manually. Values represent mean ± SD of 10 fields. *P < 0.05 versus control.

Transduction of lung epithelial cells by rAd in saline compared with rAd suspended in PFC was first examined using A549 cells, a human lung carcinoma type II cell line. rAd-CMVLacZ was added to A549 cells for 2, 4 or 8 h, and *β-*gal activity was assayed 48 h after the addition of rAd for all time points. The timeframe of A549 cell transduction was different for rAd delivery in PFC liquid compared to rAd in saline (Figure 1). Transduction in PFC liquid occurred sooner than in saline; at 2 and 4 h there were four-to-five fold more *β* gal (+) cells with PFC liquid (*P* < 0.05) compared to saline controls. By 8 h, >95% of cells in both cultures were *β-*gal (+) (data not shown).

### 3.2. PP2:PP9:rAd Suspensions Improve Distribution to Murine Distal Lung

**Figure 2:**
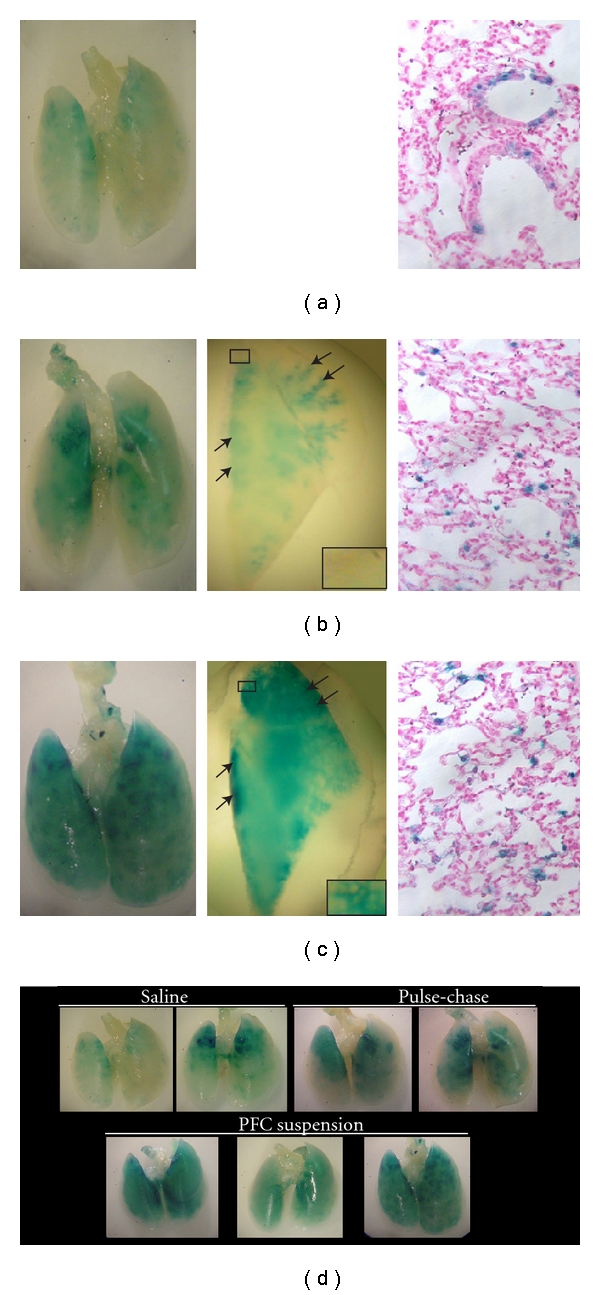
PP2/PP9 suspensions improve transgene expression in the distal lung. Left Panel: whole mounts of lungs from mice transduced with rAd-CMVLacZ (2.7 × 10^10^ viral particles) administered intratracheally in (a) saline alone, (b) saline followed by a chase of PP2:PP9 (10 mL/kg), or (c) rAd/PP2:PP9 suspension. (a) Proximal airway expression is seen predominantly when rAd is delivered in saline alone. (b) Increased expression in proximal and distal lung is seen when delivery of the virus in saline is followed by a PP2:PP9 chase; (c) Greatest staining compared to the other methodologies in the alveolar spaces and the distal lung with delivery of rAd in PP2:PP9 suspensions. Middle Panel: lungs were removed, embedded in paraffin, and sectioned to the same approximate depth. Inset in middle panel C demonstrates a distinctly alveolar pattern in the PFC liquid/rAd lungs and a lack of staining in the corresponding region of the virus in saline is followed by a PP2:PP9 chase (inset, middle panel (b)). Right Panel: sections were mounted on slides and counterstained with eosin.. When rAd is delivered in saline (right panel (a)), the signal was predominantly detected in the central airways with very little *β*-gal expression seen in the lung parenchyma. With virus delivered in saline followed by a PP2:PP9 chase (right panel (b)), there is an increase in the intensity of staining in the corresponding regions of the airways with more evidence of peripheralization With virus delivered as PFC suspension (right panel (c)), *β*-gal expression is seen in more global regions of the parenchyma. Based on the location and morphology, these cells appear to be type II cells. Panel D displays additional examples of whole mounts of lungs from mice transduced with rAd-CMVLacZ (2.7 × 10^10^ viral particles) administered in saline alone, by saline followed by a chase of PP2:PP9 (10 mL/kg), or as a rAd/PP2:PP9 suspension.

Transduction *in vivo* was tested using three different rAd-CMVLacZ administration methods: (a) in saline, (b) pulse-chase—in saline followed by PFC liquid, or (c) in PFC liquid suspension. Forty-eight hours after instillation lungs were removed en bloc and stained for *β-*gal activity (Figure 2: left panel). X-gal stained lungs were then embedded in paraffin and examined by light microscopy to compare the cell-type distribution of rAd-CMVLacZ-mediated *β-*gal expression resulting from the different methods of delivery (Figure 2: right panel). Representative lungs (Figure 2) showed that rAd-CMVLacZ delivered in saline alone (Figure 2(a)) resulted in *β-*gal activity in central and proximal large airways only. No staining was observed in the lungs of saline-alone or PFC-alone control mice (data not shown). Low magnification photos of lungs instilled by pulse-chase (Figure 2(b): middle panel) and PFC liquid suspension (Figure 2(c): middle panel) illustrated expression in large airways (see arrows). When delivered by pulse-chase method (Figure 2(b)), more cells expressed *β-*gal, and the level of expression was increased in the distal airways; however, there was little evidence of expression in the peripheral lung, especially the alveolar epithelium. In contrast, when delivered as a PFC liquid suspension (Figure 2(c)), X-gal staining was clearly more intense, more homogenous and reached the extreme peripheral lung thus reflecting the most uniform, highest, and widest distribution of *β-*gal expression. Supplementary images of lungs of mice transfected by the three techniques are shown in Figure 2(d).

*β-*gal expression in different cell types was then examined in tissue sections under higher magnification (Figure 2: right panel). Airway epithelial cells were identified by their location lining the airway. While cell-specific staining was not performed, transgene expression was evident in what appears to be alveolar epithelial cells by shape and location within alveoli; type I cells with an elongated appearance and type II cells with a cuboidal appearance are located in corners. The distribution of *β-*gal expression appeared different depending upon the method of delivery, particularly in distal portions of the lung. rAd-CMVLacZ delivery by pulse-chase appeared to result in expression primarily in airway and less so in alveoli with most alveolar expression occurring in type I cells (Figure 2(b)). In contrast, administration in PFC liquid suspension appeared to result in greater expression in alveolar epithelial cells (Figure 2(c)).

### 3.3. PP2:PP9 Improve Distribution and Early Expression of rAd

**Figure 3:**
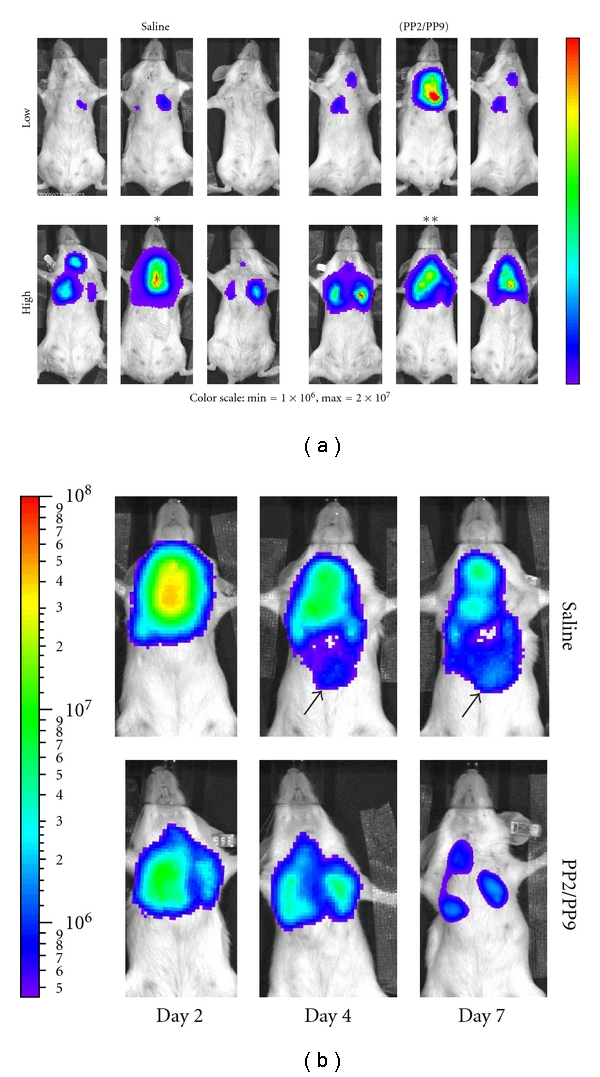
Comparison of spatial distribution of rAd-saline and -PP2/PP9 suspensions. (a) Expression at 1 d after instillation. Overlay of photographs and luminescent images of three representative animals per group. Mice were instilled with 5 × 10^10^ viral particles of rAd.luc suspended in either saline (left) or PP2:PP9 suspension (right) as described in Materials and Methods. The images were acquired 10 min after intraperitoneal delivery of luciferin (100 *μ*L; 150 mg/kg). Luminescent images were acquired under identical settings (3 min acquisition). Images from day 1 are scaled linearly with the range of signal indicated. Asterisks denote animals whose images are shown at later time points in panel (b). (b) Delivery with PP2/PP9 results in better retention in the lung. Overlay of photographs and luminescent images of representative animals from the high-dose groups is denoted in panel (a). The mice were imaged on successive days after instillation as indicated. Images are scaled logarithmically (note scale on the left).

**Figure 4:**
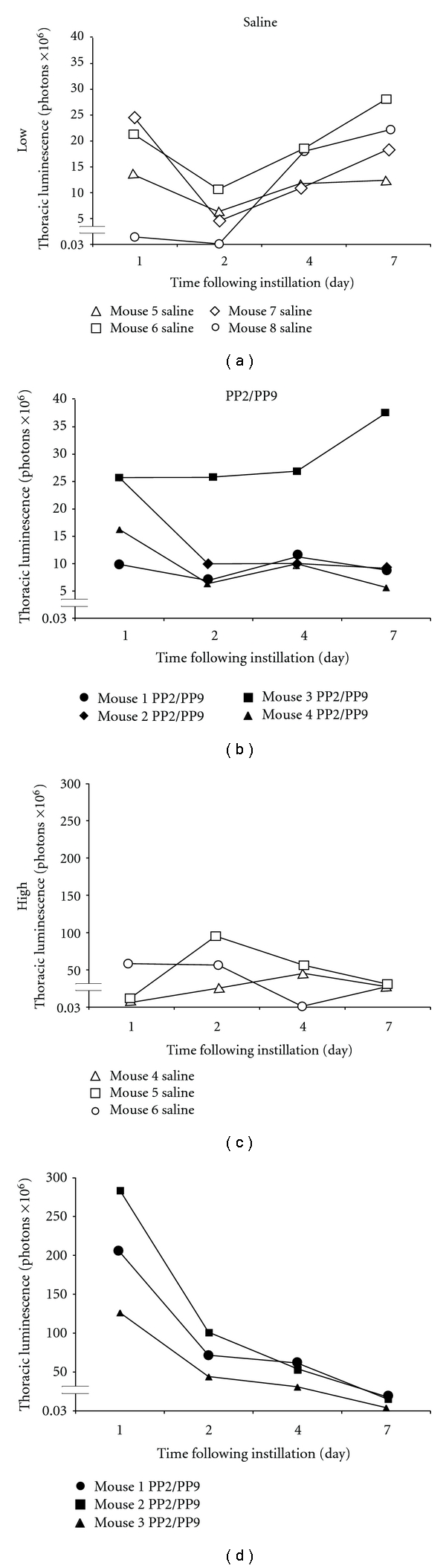
Comparison of luciferase activity in the lungs of mice. Mice were instilled with rAd-luc, and thoracic luciferase activity was quantified noninvasively over the course of 7 d. Two viral titers were administered 1 × 10^10^ viral particles (Low) or 5 × 10^10^ viral particles (High) in either saline or PFC liquid suspension. (a) 1 × 10^10^ viral particles delivered in saline; (b) 1 × 10^10^ viral particles delivered in PFC liquid; (c) 5 × 10^10^ viral particles delivered in saline; (d) 5 × 10^10^ viral particles delivered in PFC liquid. Each line represents the luciferase activity of a single animal. The images were acquired 10 min after intraperitoneal delivery of luciferin (150 mg/kg). Luminescent images were acquired under identical settings (3 min acquisition), and activity was assessed using an identical area (region of interest) and analyzed using the Living Image software package. The PFC liquid groups demonstrate a more consistent temporal pattern of expression compared to saline.

The light microscopy data demonstrate better delivery to the peripheral lung parenchyma with virus suspended in PFC. To determine if this translates into more transgene expression in the lung, we used luciferase as a reporter for two reasons: (1) transgene expression could be measured noninvasively and repeatedly over time and (2) the half-life of luciferase is short (*∼*3 h) so new transgene expression, rather than protein accumulation, is measured. In these studies, rAd-luciferase in saline or as a PFC suspension was instilled intracheally, and a longitudinal study was performed using a low-light imaging system. Balb/c mice were used for these experiments because their white coat color minimizes absorbsion of light emitted from the vector. Two viral titers were tested (1 × 10^10^ viral particles and 5 × 10^10^ viral particles); images were acquired at various times after administration, and luciferase activity was quantified. Representative animals from each group are shown in Figure 3. Animals receiving virus suspended in PFC had better bilateral distribution at both low and to a greater degree, higher viral titers (Figure 3(a)). At later times, the appearance of luciferase activity in the diaphragm area of the mice from the saline-delivery group suggests that the virus was cleared by mucociliary transport and then ingested (see arrows in Figure 3(b)). While 50% of the saline group had signal in the abdominal area, no expression was detected in the abdominal area of animals from the PFC group suggesting less clearance and better retention in the lung. Quantitation of expression was then used to compare transgene expression in the thoracic region of the mice.  Figure 4 shows the temporal pattern of individual animals, and Table 1 provides the expression when analyzed as a group. No consistent pattern was evident in the saline groups (Figures 4(a) and 4(c)) with thoracic expression peaking at 4 d, 2 d, and 1 d in Mouse 4, Mouse 5, and Mouse 6, respectively (Figure 4(c)). In the high viral titer group, animals in the PFC liquid group had a consistent pattern of expression peaking on day 1 then diminishing daily (Figure 4(d)). The PFC liquid animals in the low viral titer group (Figure 4(b)) exhibited the same pattern of expression as in the high viral titer group with one notable exception (Figure 4(b), Mouse 3). When viewed and analyzed as a group, the most dramatic differences were observed when comparing the PFC liquid and saline groups at the high viral titer (Table 1). Due to the variability in the temporal pattern of the saline group with the low viral titer (Figure 4(a)), no statistical differences were found between the PFC liquid and saline groups at any of the time points (Table 1).

## 4. Discussion

This study demonstrates that a specific combination of PFC liquids (PP2:PP9), that we have previously shown to significantly improve gas exchange, lung function, and attenuate inflammation in animal models of lung injury [13, 15, 35], also significantly improves the efficiency of rAd gene delivery to the peripheral lung. The generation of PFC liquid/rAd suspensions specifically improved expression in the alveolar epithelial cells. 

Recombinant adenovirus can induce high levels of transient transgene expression. Several important improvements have been made in this vector resulting in enhanced gene delivery to the lung. One of earliest improvements was to change from serotype 7 to serotype 5. Since serotype 5 binds the CAR receptor found in airways more efficiently, the viral titer required for gene expression is attenuated, and inflammation significantly reduced. Next, reagents such as EGTA that disrupt tight junctions have been shown to improve expression in the airways by exposing CAR on the basolateral surfaces of airway epithelium. Various PFC liquids have been utilized to improve expression in the distal airways of both healthy and diseased lungs [3, 6–10]. Our findings reflect at least two mechanisms thought to be involved with improved expression. First, it has been previously shown that PFC liquid facilitates transient disruption of tight junctions, increasing exposure to CARs, that are distributed throughout the A549 cell membrane [3]. Second, based on the physicochemical property of this specific PFC liquid combination (with unique kinematic viscosity, vapor pressure, and lipophilicity profiles) [14, 36], the PFC suspension fosters increased and uniformed distribution of the virus relative to saline, such that more cells are exposed to virus. As such, clinical use of PFC liquids for the delivery of rAd could reduce the amount of virus necessary for the treatment of lung disease. This possibility is an important consideration given the inherent inflammatory response associated with adenovirus. The unique physicochemical properties of the PFC suspension may support further improvement over viscoelastic gels which have been recently shown to improve rAd and AAV expression only in the central airways without increases in alveolar expression [37]. Our data document significant differences in transgene expression with rAd delivered in PFC versus saline at early time points both *in vitro* and *in vivo*. *In vitro* we demonstrate a higher earlier transduction efficiency and *in vivo* a more consistent pattern of temporal expression. We have demonstrated the effect of different PFC liquids on membrane fluidity and packaging using liposomes as model systems [28]. When the amount of luciferase activity was assessed in the present study, significant differences in expression were only detected at 1 d following administration when PFC liquid suspensions were compared to other methods. We speculate that temporal differences between methods of administration (favoring PFC suspension) may actually be greater than the present analysis; however, we believe that more detailed analyses may be confounded by the high level of focal expression in upper airways with saline instillation as compared to the more peripheral distribution with PFC suspension. As such, this report demonstrates that the major advantages of PFC liquid-suspension delivery of rAd are increased early expression (Figure 4, Table 1), better distal lung expression, and better retention in the lung (Figure 3). 

While this study was not designed to comprehensively assess ectopic expression, our method of introduction by minitracheal puncture is likely to oppose this possibility since this method bypasses the nasal epithelium as well as minimizes the potential for the virus to move up the mucocililiary tract to transduce the gut. In addition, the alveolar surfaces appeared intact thereby minimizing access for ectopic expression through translocation across the alveolar-capillary membrane. In addition, there was very little ectopic expression detected with the luciferase construct alone. 

Previous studies using PFC liquid vehicles for rAd-gene delivery utilized a pulse-chase method of administration, that is, delivery of rAd in saline followed immediately by instillation of PFC liquid [7, 9, 11, 38]. We reasoned that creating a suspension should increase efficiency by increasing the distribution of the vector compared to the pulse-chase method. We have demonstrated previously that formation of nanocrystal suspensions with PFC liquids improves delivery of biological agents (recombinant proteins and antibiotics) to the lung [33, 35, 39]. However this technology is dependent on the ability to form powders of the drug/protein of interest. Since rAd is relatively labile and loses viability when lyophilized, we limited our methods to creating liquid suspensions. There have been reports of formulations that stabilize rAd for freeze-dry methods, but our attempts to create nanocrystals using this particular method proved unsuccessful (data not shown). More stable gene delivery vectors or compounds (plasmid DNA, rSV40, siRNA) would be viable candidates for PFC liquid nanocrystal suspension formation. The efficacy of siRNA strategies, delivered as either oligonucleotides or in other gene vector systems, provides the possibility of suppressing gene expression.

No method of gene delivery is ideal, and there are significant problems associated with the use of rAd (e.g., inflammation; cell specificity limited to cells expressing the appropriate receptor for viral entry), especially in the clinical setting. Several strategies have been implemented to try to minimize these problems, including generating “gutted virus” [40] and the use of chimeric fiber [41, 42]. In spite of the potential difficulties of rAd, these vectors still hold promise for delivering transient overexpression of genes. As demonstrated in the present study, PFC liquid promotes uniform delivery to the lung and may minimize the inflammatory response to rAd gene products [9]. This would be particularly important in nonhomogeneous and inflammatory types of lung injury processes where PFC liquids could facilitate distribution of genes or other biologic agents while facilitating gas exchange and improving pulmonary function for management/treatment (i.e., cystic fibrosis, COPD, or ARDS). In contrast, in disease states that affect the central or proximal airways, delivery by saline or the pulse-chase method might be advantageous.

The PFC liquid formulation used in the present studies was selected based on the greatest improvement in gas exchange and lung mechanics and attenuation of lung inflammation in models of acute lung injury [13, 15, 35]. Within this context, the use of PFC suspensions to enhance gene expression challenges the notion that minor inflammation is required for gene uptake. The major improvement in gene expression *in vivo *appears to be in the delivery of the virus to alveolar epithelial cells of the distal lung. Why this increase is observed in these cell types remains unclear. The most likely explanation is that the specific physicochemical properties (e.g., kinematic viscosity, vapor pressure, and lipophilic nature) of PFC liquid enhance delivery to and assist cells in the transduction of the alveoli (i.e., similar to how surfactant is recycled, alterations in membrane dynamics). We suggest that delivery into this compartment prevents clearance by the mucociliary tract. 

In summary, PP2:PP9 PFC rAd suspensions increased expression in distal portions of the lung. The suspensions may facilitate receptor-mediated and independent entry of the virus which significantly increases transduction efficiency. This has important therapeutic implications for diseases where expression in the central airways is not sufficient and expression in the alveoli is required. The use of PFC liquid holds promise as a vehicle to deliver gene delivery vectors and other biologic agents in order to more directly and specifically prevent and treat high-risk lung disease processes in critically ill patients. Others have implicated a “surfactant” mediated uptake of PFC into alveolar type II cells, ostensibly associated with an endocytotic process [31, 36, 43]. If these particles are then secreted out into the lung surface, it would foster recycling of the virus to transduce additional or adjacent alveolar epithelial cells. If this theory holds true, the use of PFC suspensions for gene vector delivery prevents a novel and forward-thinking contribution to the field of gene therapy. Further study of PFC-assisted gene vector delivery methods, including nebulization of PFC liquid suspensions, is warranted to potentially overcome many of obstacles currently associated with gene delivery to the lung epithelium.

## Figures and Tables

**Table 1:** Temporal pattern of thoracic luciferase expression.

Vehicle	Viral load (viral particles)	Days after instillation			
		1	2	4	7
PP2:PP9	5 × 10^10^	4,021 ± 950	1,127 ± 275	822 ± 100	227 ± 37
Saline	5 × 10^10^	460 ± 665	2,246 ± 2,787	707 ± 675	453 ± 185
PP2:PP9	1 × 10^10^	988 ± 1,237	278 ± 228	313 ± 213	283 ± 299
Saline	1 × 10^10^	232 ± 167	79 ± 63	194 ± 43	260 ± 60

Animals were instilled with either 5 × 10^10^ viral particles (*n* = 3/group) or 1 × 10^10^ viral particles (*n* = 4/group) rAd-Luc intratracheally using either saline or as a PP2:PP9 PFC suspension. Mice were anesthetized, and images were acquired at the time indicated using a cooled CCD camera and imaging system (IVIS50, Xenogen Corp). Luminescence was quantified using Living Image software with a constant region of interest. Values represent the mean ± standard deviation.

## References

[1] Kolb M, Martin G, Medina M, Ask K, Gauldie J (2006). Gene therapy for pulmonary diseases. *Chest*.

[2] Pickles RJ (2004). Physical and biological barriers to viral vector-mediated delivery of genes to the airway epithelium. *Proceedings of the American Thoracic Society*.

[3] Weiss DJ, Beckett T, Bonneau L, Young J, Kolls JK, Wang G (2003). Transient increase in lung epithelial tight junction permeability: an additional mechanism for enhancement of lung transgene expression by perfluorochemical liquids. *Molecular Therapy*.

[4] Chu Q, St. George JA, Lukason M, Cheng SH, Scheule RK, Eastman SJ (2001). EGTA enhancement of adenovirus-mediated gene transfer to mouse tracheal epithelium in vivo. *Human Gene Therapy*.

[5] Wang G, Zabner J, Deering C (2000). Increasing epithelial junction permeability enhances gene transfer to airway epithelia in vivo. *American Journal of Respiratory Cell and Molecular Biology*.

[6] Li JT, Bonneau LA, Zimmerman JJ, Weiss DJ (2007). Perfluorochemical (PFC) liquid enhances recombinant adenovirus vector-mediated viral interleukin-10 (AdvIL-10) expression in rodent lung. *Journal of Inflammation*.

[7] Weiss DJ, Strandjord TP, Liggitt D, Clark JG (1999). Perflubron enhances adenovirus-mediated gene expression in lungs of transgenic mice with chronic alveolar filling. *Human Gene Therapy*.

[8] Weiss DJ, Strandjord TP, Jackson JC, Clark JG, Liggitt D (1999). Perfluorochemical liquid-enhanced adenoviral vector distribution and expression in lungs of spontaneously breathing rodents. *Experimental Lung Research*.

[9] Weiss DJ, Bonneau L, Liggitt D (2001). Use of perfluorochemical liquid allows earlier detection of gene expression and use of less vector in normal lung and enhances gene expression in acutely injured lung. *Molecular Therapy*.

[10] Weiss DJ, Baskin GB, Shean MK, Blanchard JL, Kolls JK (2002). Use of perflubron to enhance lung gene expression: safety and initial efficacy studies in non-human primates. *Molecular Therapy*.

[11] Lisby DA, Ballard PL, Fox WW, Wolfson MR, Shaffer TH, Gonzales LW (1997). Enhanced distribution of adenovirus-mediated gene transfer to lung parenchyma by perfluorochemical liquid. *Human Gene Therapy*.

[12] Cox CA, Stavis RL, Wolfson MR, Shaffer TH (2003). Long-term tidal liquid ventilation in premature lambs: physiologic, biochemical and histological correlates. *Biology of the Neonate*.

[13] Jeng MJ, Yang SS, Wolfson MR, Shaffer TH (2003). Perfluorochemical (PFC) combinations for acute lung injury: an in vitro and in vivo study in juvenile rabbits. *Pediatric Research*.

[14] Miller TF, Milestone B, Stern R, Shaffer TH, Wolfson MR (2001). Effects of perfluorochemical distribution and elimination dynamics on cardiopulmonary function. *Journal of Applied Physiology*.

[15] Shashikant BN, Miller TL, Jeng MJ, Davis J, Shaffer TH, Wolfson MR (2005). Differential impact of perfluorochemical physical properties on the physiologic, histologic, and inflammatory profile in acute lung injury. *Critical Care Medicine*.

[16] Stavis RL, Wolfson MR, Cox CA, Kechner N, Shaffer TH (1998). Physiologic, biochemical, and histologic correlates associated with tidal liquid ventilation. *Pediatric Research*.

[17] Wolfson MR, Kechner NE, Roache RF (1998). Perfluorochemical rescue after surfactant treatment: effect of perflubron dose and ventilatory frequency. *Journal of Applied Physiology*.

[18] Wolfson MR, Greenspan JS, Deoras KS, Rubenstein SD, Shaffer TH (1992). Comparison of gas and liquid ventilation: clinical, physiological, and histological correlates. *Journal of Applied Physiology*.

[19] Rotta AT, Gunnarsson B, Hernan LJ, Fuhrman BP, Steinhorn DM (2000). Partial liquid ventilation with perflubron attenuates in vivo oxidative damage to proteins and lipids. *Critical Care Medicine*.

[20] Steinhorn DM, Papo MC, Rotta AT, Aljada A, Fuhrman BP, Dandona P (1999). Liquid ventilation attenuates pulmonary oxidative damage. *Journal of Critical Care*.

[21] Croce MA, Fabian TC, Patton JH, Melton SM, Moore M, Trenthem LL (1998). Partial liquid ventilation decreases the inflammatory response in the alveolar environment of trauma patients. *Journal of Trauma*.

[22] Wolfson MR, Shaffer TH (2004). Liquid ventilation: an adjunct for respiratory management. *Paediatric Anaesthesia*.

[23] Wolfson MR, Shaffer TH (2005). Pulmonary applications of perfluorochemical liquids: ventilation and beyond. *Paediatric Respiratory Reviews*.

[24] Varani J, Hirschl RB, Dame M, Johnson K (1996). Perfluorocarbon protects lung epithelial cells from neutrophil-mediated injury in an in vitro model of liquid ventilation therapy. *Shock*.

[25] Obraztsov VV, Neslund GG, Kornbrust ES, Flaim SF, Woods CM (2000). In vitro cellular effects of perfluorochemicals correlate with their lipid solubility. *American Journal of Physiology*.

[26] Woods CM, Neslund G, Kornbrust E, Flaim SF (2000). Perflubron attenuates neutrophil adhesion to activated endothelial cells in vitro. *American Journal of Physiology*.

[27] Nakstad B, Wolfson MR, Shaffer TH (2001). Perfluorochemical liquids modulate cell-mediated inflammatory responses. *Critical Care Medicine*.

[28] Venegas B, Wolfson MR, Cooke PH, Chong PLG (2008). High vapor pressure perfluorocarbons cause vesicle fusion and changes in membrane packing. *Biophysical Journal*.

[29] Gabriel JL, Miller TF, Wolfson MR, Shaffer TH (1996). Quantitative structure-activity relationships of perfluorinated hetero- hydrocarbons as potential respiratory media: application to oxygen solubility, partition coefficient, viscosity, vapor pressure, and density. *ASAIO Journal*.

[30] Shaffer TH, Foust R, Wolfson MR, Miller TF (1997). Analysis of perfluorochemical elimination from the respiratory system. *Journal of Applied Physiology*.

[31] Wemhöner A, Hackspiel I, Hobi N, Ravasio A, Haller T, Rüdiger M (2010). Effects of perfluorocarbons on surfactant exocytosis and membrane properties in isolated alveolar type II cells. *Respiratory Research*.

[32] Davidson BL, Allen ED, Kozarsky KF, Wilson JM, Roessler BJ (1993). A model system for in vivo gene transfer into the central nervous system using an adenoviral vector. *Nature Genetics*.

[33] Cox CA, Cullen AB, Wolfson MR, Shaffer TH (2001). Intratracheal administration of perfluorochemical-gentamicin suspension: a comparison to intravenous administration in normal and injured lungs. *Pediatric Pulmonology*.

[34] Cullen AB, Cox CA, Hipp SJ, Wolfson MR, Shaffer TH (1999). Intra-tracheal delivery strategy of gentamicin with partial liquid ventilation. *Respiratory Medicine*.

[35] Sarafidis K, Malone DJ, Zhu G (2008). Perfluorochemical augmented rhSOD delivery attenuates inflammation in the immature lung. *Journal of Neonatal-Perinatal Medicine*.

[36] Rüdiger M, Wendt S, Köthe L, Burkhardt W, Wauer RR, Ochs M (2007). Alterations of alveolar type II cells and intraalveolar surfactant after bronchoalveolar lavage and perfluorocarbon ventilation. An electron microscopical and stereological study in the rat lung. *Respiratory Research*.

[37] Sinn PL, Shah AJ, Donovan MD, McCray PB (2005). Viscoelastic gel formulations enhance airway epithelial gene transfer with viral vectors. *American Journal of Respiratory Cell and Molecular Biology*.

[38] Strayer MS, Guttentag SH, Ballard PL (1998). Targeting type II and Clara cells for adenovirus-mediated gene transfer using the surfactant protein B promoter. *American Journal of Respiratory Cell and Molecular Biology*.

[39] Brunelli L, Hamilton E, Davis JM (2006). Perfluorochemical liquids enhance delivery of superoxide dismutase to the lungs of juvenile rabbits. *Pediatric Research*.

[40] Cao H, Koehler DR, Hu J (2004). Adenoviral vectors for gene replacement therapy. *Viral Immunology*.

[41] Hedley SJ, Auf der Maur A, Hohn S (2006). An adenovirus vector with a chimeric fiber incorporating stabilized single chain antibody achieves targeted gene delivery. *Gene Therapy*.

[42] Koizumi N, Mizuguchi H, Kondoh M, Fujii M, Hayakawa T, Watanabe Y (2004). Efficient gene transfer into human trophoblast cells with adenovirus vector containing chimeric type 5 and 35 fiber protein. *Biological and Pharmaceutical Bulletin*.

[43] Steinhorn DM, Papo MC, Aljada A, Thusu K, Fuhrman BP, Dandonna P (1996). Partial liquid ventilation (PLV) with perflubron decreases oxidative damage to the lung during experimental injury. *Critical Care Medicine*.

